# Risk Management Framework for Nano-Biomaterials Used in Medical Devices and Advanced Therapy Medicinal Products

**DOI:** 10.3390/ma13204532

**Published:** 2020-10-13

**Authors:** Elisa Giubilato, Virginia Cazzagon, Mónica J. B. Amorim, Magda Blosi, Jacques Bouillard, Hans Bouwmeester, Anna Luisa Costa, Bengt Fadeel, Teresa F. Fernandes, Carlos Fito, Marina Hauser, Antonio Marcomini, Bernd Nowack, Lisa Pizzol, Leagh Powell, Adriele Prina-Mello, Haralambos Sarimveis, Janeck James Scott-Fordsmand, Elena Semenzin, Burkhard Stahlmecke, Vicki Stone, Alexis Vignes, Terry Wilkins, Alex Zabeo, Lang Tran, Danail Hristozov

**Affiliations:** 1 Department of Environmental Sciences, Informatics and Statistics, University Ca’ Foscari of Venice, Via Torino 155, 30172 Venice, Italy; giubilato@unive.it (E.G.); virginia.cazzagon@unive.it (V.C.); marcom@unive.it (A.M.); semenzin@unive.it (E.S.); 2 Department of Biology and CESAM, University of Aveiro, 3810-193 Aveiro, Portugal; mjamorim@ua.pt; 3 Institute of Science and Technology for Ceramics, National Research Council of Italy (CNR-ISTEC), Via Granarolo 64, 48018 Faenza, Italy; magda.blosi@istec.cnr.it (M.B.); anna.costa@istec.cnr.it (A.L.C.); 4 Institut National de l’Environnement industriel et des Risques, Parc Technologique ALATA, 60550 Verneuil-en-Halatte, France; Jacques.bouillard@ineris.fr (J.B.); alexis.vignes@ineris.fr (A.V.); 5 Division of Toxicology, Wageningen University, 6708 WE Wageningen, The Netherlands; hans.bouwmeester@wur.nl; 6 Division of Molecular Toxicology, Institute of Environmental Medicine, Karolinska Institutet, 171 77 Stockholm, Sweden; bengt.fadeel@ki.se; 7 Institute of Life and Earth Sciences, School of Energy, Geoscience, Infrastructure and Society, Heriot-Watt University, Edinburgh EH14 4AS, UK; T.Fernandes@hw.ac.uk; 8 Instituto Tecnologico del Embalaje, Transporte y Logistica, 46980 Paterna-Valencia, Spain; carlos.fito@itene.com; 9 Empa, Swiss Federal Laboratories for Materials Science and Technology, Lerchenfeldstrasse 5, 9014 St. Gallen, Switzerland; Marina.Hauser@empa.ch (M.H.); nowack@empa.ch (B.N.); 10 GreenDecision Srl, Via delle Industrie, 21/8, 30175 Venice, Italy; lisa.pizzol@greendecision.eu (L.P.); alex.zabeo@greendecision.eu (A.Z.); 11 Institute of Biological Chemistry, Biophysics and Bioengineering, School of Engineering and Physical Sciences, Heriot-Watt University, Edinburgh EH14 4AS, UK; l.powell@hw.ac.uk (L.P.); v.stone@hw.ac.uk (V.S.); 12 Trinity Translational Medicine Institute, Trinity College, The University of Dublin, Dublin 8, Ireland; prinamea@tcd.ie; 13 School of Chemical Engineering, National Technical University of Athens, 15780 Athens, Greece; hsarimv@central.ntua.gr; 14 Department of Bioscience, Aarhus University, 8600 Silkeborg, Denmark; jsf@bios.au.dk; 15 Institut für Energie und Umwelttechnik e.V., 47229 Duisburg, Germany; stahlmecke@iuta.de; 16 Nanomanufacturing Institute, School of Chemical and Process Engineering, University of Leeds, Leeds LS2 9JT, UK; t.a.wilkins@leeds.ac.uk; 17 Institute of Occupational Medicine, Research Avenue North, Riccarton, Edinburgh EH14 4AP, UK; lang.tran@iom-world.org

**Keywords:** risk management, nano-biomaterials, nanomedicine, medical device, life cycle, safe-by-design

## Abstract

The convergence of nanotechnology and biotechnology has led to substantial advancements in nano-biomaterials (NBMs) used in medical devices (MD) and advanced therapy medicinal products (ATMP). However, there are concerns that applications of NBMs for medical diagnostics, therapeutics and regenerative medicine could also pose health and/or environmental risks since the current understanding of their safety is incomplete. A scientific strategy is therefore needed to assess all risks emerging along the life cycles of these products. To address this need, an overarching risk management framework (RMF) for NBMs used in MD and ATMP is presented in this paper, as a result of a collaborative effort of a team of experts within the EU Project BIORIMA and with relevant inputs from external stakeholders. The framework, in line with current regulatory requirements, is designed according to state-of-the-art approaches to risk assessment and management of both nanomaterials and biomaterials. The collection/generation of data for NBMs safety assessment is based on innovative integrated approaches to testing and assessment (IATA). The framework can support stakeholders (e.g., manufacturers, regulators, consultants) in systematically assessing not only patient safety but also occupational (including healthcare workers) and environmental risks along the life cycle of MD and ATMP. The outputs of the framework enable the user to identify suitable safe(r)-by-design alternatives and/or risk management measures and to compare the risks of NBMs to their (clinical) benefits, based on efficacy, quality and cost criteria, in order to inform robust risk management decision-making.

## 1. Introduction

The convergence of nanotechnology and biotechnology has created huge potential for advancements in medical diagnosis, therapy and regenerative medicine, which has fostered large investments in developing novel nano-biomaterials (NBMs) for use in medical devices (MD) and in medicinal products, including advanced therapy medicinal products (ATMP) (cf. definitions in SI Section 1). NBMs are a special category of biomaterials, which possess a constituent or have a surface dimension in the nano range (i.e., 1–100 nm) [1]. These new materials can offer unprecedented technological benefits as their interactions with the biological systems can improve biocompatibility as well as clinical efficacy, while reducing adverse effects [2,3,4,5]. However, because of their more complex nature and nano-bio/eco interactions (i.e., interactions of the nano-sized component with biological molecules and structures, and with the surrounding environmental media in case of release in the environment), there are both scientific and societal concerns about their possible health and/or environmental risks. These concerns are magnified by the lack of fundamental research on the interactions of NBM with human physiological processes (in both patients and other users), and the interactions that NBM can have upon release into the environment during the product life cycle. In addition, there is a concern on the adequacy of regulatory guidance addressing the complexity of these materials. Today we face the issue that the research and development (R&D) into nanobiomedical innovation has progressed faster than the generation of adequate tools to assess the risk-benefit balance of these technologies. It is therefore challenging to propose effective risk management strategies in accordance with the regulatory requirements legally enforced to ensure their safety, efficacy and quality [6,7,8,9].

While a robust assessment of the safety of the NBM-based medical applications for the patients is a prerequisite for regulatory approval [10], the assessment and management of the possible occupational and/or environmental risks resulting from unintentional exposure to these materials are not strictly required by the European Medicines Agency (EMA) and the national medicinal regulations, and are therefore often overlooked. 

To address this issue, the main goal of this paper is to propose an overarching framework for risk management of NBMs used in MD and ATMP, which complements the preclinical benefit-risk analysis of these technologies with a complete assessment of their risks for the environment as well as for medical professionals (e.g., physicians, technicians, nurses and healthcare assistants) and workers exposed to NBMs during production (e.g., powder/liquid handling), use (e.g., abrasion, leaching) and/or end-of-life treatment (e.g., disposal, incineration) (a list of industries producing NBMs or NMB-based MD and ATMP, as well as healthcare facilities where they can be used is provided in Table S1). This approach has been developed within the EU Horizon 2020 project BIORIMA (BIOmaterials RIsk MAnagement; Grant Agreement No: 760928), which has built upon the results of many EU FP7 and Horizon 2020 projects with the aim to adapt the knowledge, data and tools for engineered nanomaterials (ENMs) to the NBMs used in MD and ATMP. 

The BIORIMA risk management framework (RMF) is the outcome of the interdisciplinary, collaborative effort of a team of experts from different European universities, research institutes and companies involved as partners in the BIORIMA project [11]. This work is based on a comprehensive review of regulatory requirements and research trends related to the risk management of nano(bio)medical technologies, which was performed with the aim of collecting information and data to inform a subsequent expert discussion. Selected experts from the BIORIMA consortium formed three working groups, namely (a) human health and ecological risk assessment, (b) benefit-risk analysis, (c) risk prevention, control and monitoring, depending on their field of expertise, and each group worked on developing/describing the corresponding parts of the framework. The draft framework was presented to stakeholders during the 1st BIORIMA Stakeholder Workshop held in Valencia in November 2018 that brought together more than 40 participants from industry, research and regulatory institutions (cf. Table S2). Workshop participants were asked to comment on the proposed framework through guided discussions during three break-out sessions and a final plenary discussion and this resulted in constructive feedback by the stakeholders, which was incorporated in the current version of the RMF, presented here. 

The goal has been to develop an RMF that is applicable to past and current generation of NBMs but at the same time is able to integrate new scientific outcomes to address the need of future generations of NBMs and to support the standardisation and regulation of these materials. 

The present paper describes the BIORIMA RMF from a conceptual standpoint. First, we describe the process and provide an overview of the positioning of its main elements, which are then described in more detail in subsequent sections. The purpose of this paper is to provide an ‘early view’ of an evolving RMF for MD and ATMP.

## 2. The BIORIMA Risk Management Framework

### 2.1. An Overview of the Risk Management Framework for NBMs

**Figure 1:**
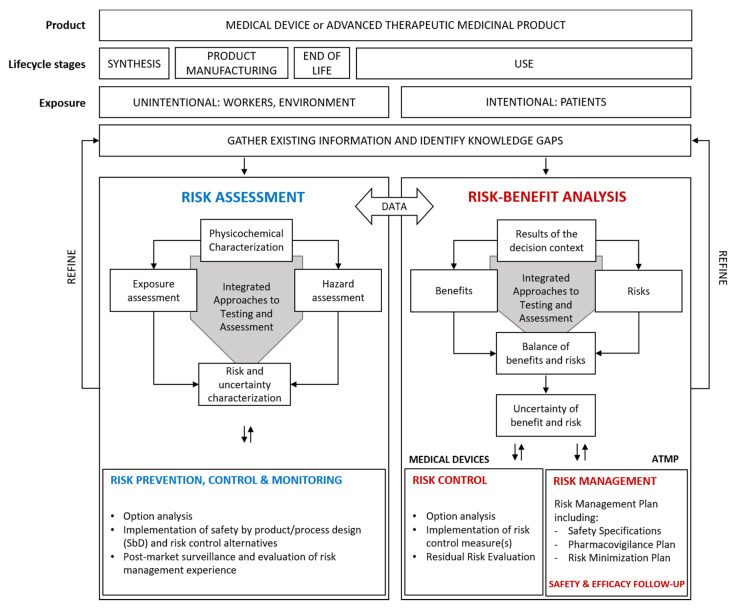
BIORIMA risk management framework for nano-biomaterials (NBMs) used in medical devices (MD) and advanced therapy medicinal products (ATMP).

The RMF is outlined in Figure 1. It is designed to facilitate risk-benefit analysis of NBMs applied in MD and ATMP for patients, as well as assessment of their occupational and environmental risks from a life cycle perspective. In cases of unacceptable risks, the RMF supports the identification of adequate risk control measures. The life cycle stages are not listed in the typical order (i.e., synthesis > formulation > use > end-of-life treatment), but rather according to different exposure assessment targets: on the left side the stages where unintentional exposure of workers or environmental targets can occur, including unintentional exposure of medical professionals; on the right side the use stage where patients are intentionally exposed for therapeutic or diagnostic purposes.

The framework starts with the choice of the type of product, i.e., a MD or an ATMP. The framework inputs include information on relevant life cycle stages of such products. Early life cycle stages such as synthesis and product manufacturing are most relevant in an occupational risk assessment, while during the use stage the risk assessment is relevant to both patients and workers (e.g., doctors, nurses, dentists). Following use, end-of life is most relevant to environmental risk assessment. The RMF provides two pillars to structure the risk assessment and management process: one relevant to occupational and environmental risks associated with unintentional exposure (left side), one relevant to the risk-benefit analysis of patients (right side). 

The regulatory framework to address occupational health and safety and environmental risks of NBMs incorporated in MD and ATMP is built upon the provisions of REACH (Registration, Evaluation, Authorisation and restriction of Chemicals) regulation, Environmental Health & Safety regulations and the associated guidance documents (e.g., REACH 2006 [12], documents supporting Directive 1999/92/CE [13] concerning Equipment intended for use in EXplosive ATmospheres, known as ATEX Directive). For workers, both accidental and chronic risks need to be considered. In an accident risk scenario, a potential hazard (e.g., explosion, fire, massive release) can impact a worker over a short period of time (an accident). This is different from the exposure frequency and duration paradigm used to estimate chronic risks and therefore requires a different approach (as described in Section 2.2.3). Once all health and environmental risks are quantified and evaluated, the outputs of the RMF include identification of appropriate approaches for risk prevention and control (cf. Section 2.3). 

The right-side pillar of the RMF focuses on the use of a NBM-based MD or ATMP for diagnostic and/or therapeutic purposes, where the efficacy and the safety of these materials/products for the patients constitute the main concern and are assessed and weighed by means of benefit-risk analysis (Section 2.3.4) [14]. In order to obtain market authorisation, benefits and risks are investigated through the execution of pre-clinical and clinical assessments, according to the current EU regulations for the commercial approval of MD and ATMP (e.g., Regulation (EU) 1394/2007 [15], Regulation (EU) 2017/74 [16], Directive 2009/120/EC [17]). Once on the market, adequate risk control measures must be considered for medical devices (following ISO 14971 [18] provisions), while for ATMP current EU legislation asks for the implementation of a risk management plan and a safety and efficacy follow-up. 

The two pillars are not disconnected. Instead a flux of information and data is recommended in order to avoid duplication of efforts that can incur additional costs. For example, data on the intrinsic physicochemical properties of NBMs are relevant to any kind of appraisal of adverse effects or health benefits and, as such, once collected or generated they should be shared and exploited within both assessment processes regardless of the regulatory regime. The same holds true for the pre-clinical toxicological studies that are required for the hazard identification and the hazard characterisation of the NBMs.

In the paragraphs above, the RMF has been presented from a life cycle perspective, however it is worth considering that the strategies composing the framework can play different roles and can be applied at different levels of detail across the stages of the innovation process. The R&D phases of a medicinal product include the non-clinical discovery phase (identification of target and preliminary candidates, pre-clinical studies for the optimisation of candidates and selection of a drug candidate for clinical testing), the clinical phases (phase I, II, III) and the post-marketing pharmacovigilance [19,20]. In the early non-clinical discovery phase industries typically rely on existing data and/or less expensive screening-level assessments by means, for example, of in silico modelling or in vitro testing approaches. For instance, data read-across from similar materials or applications can be used to identify suitable candidates for further testing, or to remove potentially toxic materials from a candidate shortlist. Later, benefit-risk analysis methods are applied to support regulatory decisions about authorisation of new MD or ATMP, when they are used to integrate data gathered through pre-clinical tests and clinical trials. In the post-marketing stage, benefit-risk analysis may be required again to weigh and integrate evidence provided by pharmacovigilance of new products. In general, approaches and methods used for risk assessment and management and benefit-risk analysis could be used in an iterative way along the innovation processes, providing at each stage new information which can support further development and assessment of the product and its production processes. 

The conceptual strategies for the assessment and management of risks associated with NBMs used in MD and ATMP proposed in the BIORIMA RMF are described in detail in the following paragraphs.

### 2.2. Risk Assessment Strategy

We have adopted strategies for human health and environmental risk assessment of the NBMs from a life cycle perspective, considering the provisions of EU regulations and using state-of-the-art scientific approaches for safety assessment of nanomaterials. These strategies are intended to support stakeholders (e.g., regulators, industries, consultants) in identifying and applying the most appropriate methods and tools (e.g., standards, testing protocols, predictive models) to assess potential risks associated with unintentional exposure of workers involved in manufacturing, use and disposal of nano-enabled MDs and ATMPs, as well as healthcare professionals exposed to NBMs while using the products. In addition, the strategy is aimed at guiding the identification of releases of NBMs into environmental compartments (i.e., air, soil, water, sediment) and the implementation of the most appropriate experimental and modelling tools to enable the assessment of their behaviour and fate (e.g., bio-persistence, bio-transformation) as well as their short and long-term toxicity effects for aquatic and terrestrial biota. 

However, at several levels there is high complexity associated with the hazard assessment of NBM, both for human risks as well as for the environment [21,22]. Common issues are dealing with the physicochemical identity of the NBMs, the existence of different nanoforms and their transformations in physiological and environmental media (e.g., biocorona formation, weathering/ageing) [23,24,25]. In addition, it can be questioned which are the most relevant biomarkers for NBM hazard testing and whether they are currently adequately addressed in the Organisation for Economic Co-operation and Development (OECD) guidelines used for regulatory purposes. 

Moreover, there are many possible human and environmental exposure scenarios that should be investigated. Therefore, a strategic approach is required to streamline, optimise and properly target the available resources using either an intelligent testing strategy [26], also known as an Integrated Approach to Testing and Assessment (IATA) [27], specifically developed for NBMs used in MDs and ATMPs. Indeed, in order to optimise the selection of the most suitable methods and guide the identification and characterisation of human and ecological risks, the risk assessment strategy proposed in the BIORIMA RMF includes a set of IATA. 

The IATA are also strongly influenced by the type of NBM to be investigated as well as the exposure route. The exposure/administration route and frequency determine important parameters such as relevant timepoints, dispersant/matrix and controls to be used in experimental testing. Each IATA is therefore tailored to address the physicochemical characteristics of the NBM, the likely route(s) of exposure or environmental compartments, the frequency of exposure and the needs of the risk assessor (e.g., regulator or developer during early innovation). 

The BIORIMA IATAs follow the structure first recommended by the OECD [28] and further developed/applied within the Horizon 2020 project GRACIOUS (“Grouping, Read-Across, CharacterIsation and classificatiOn framework for regUlatory risk assessment of manufactured nanomaterials and Safer design of nano-enabled products”) [29]. The IATAs take the form of decision trees, structured to collect the information and data needed for human health and ecological risk assessment of NBMs. Each question (or decision node) within the decision tree prompts the user to strategically select the most appropriate testing or alternative methods (or a combination) for hazard assessment. These include in silico (e.g., physiologically-based pharmaco-kinetic (PBPK) models), in vitro, ex vivo and in vivo approaches. The IATA have been developed to utilise, as far as possible, existing data (by utilising data in open-access databases), non-testing approaches (e.g., in silico) and other alternatives to animal testing (e.g., in vitro). This is achieved by employing a tiered testing strategy to address each decision node, in which the complexity of the model employed increases from tier 1 to 3. 

Before initiating any testing by means of the IATA, a review of the available data is required to identify areas of immediate concerns regarding the NBMs toxic potency and/or exposure potential. This includes information from clinical studies (preferably), then human relevant in vitro and in silico data, and lastly animal in vivo data. This analysis also allows a gap analysis of the hazard data relevant to that specific NBM. The missing data are then generated by employment of the IATA. For human hazard assessment, tier 1 focuses on in silico, in chemico and simple (one cell type monoculture) in vitro models, tier 2 includes more complex alternative approaches such as multi-cell lineage three dimensional co-cultures (e.g., organoids, organ-on-chip) and tier 3 is largely based on animal models including hazard and biodistribution testing. The in vivo data currently remains vital for the NBMs to progress to clinical trials, and also for their full occupational risk assessment. However, the results of tier 1 and 2 are used to refine the animal studies in terms of the most relevant concentrations, timepoints and endpoints to assess, thereby reducing the number of animals used. The IATAs therefore optimise the cost of obtaining relevant information and data, while reducing the use of experimental animals in accordance with the 3Rs (replacement, reduction and refinement) principles [30].

The tiers are useful since the nature and level of information needed to support product development decisions is different from the data needed for regulatory approval. Therefore, the type and extent of testing can be varied according to the actual purpose of the assessment. 

#### 2.2.1. Occupational Risk Assessment

In the European Union the occupational safety of NBMs is regulated by the Commission Regulation 2018/1881 [31] which modified Annexes I, III and VI-XII of the REACH (regulation, introducing specific requirements and guidelines to cover nanoforms. These requirements are of course also applicable to the NBMs used in medicine. REACH requires Chemical Safety Assessment for each substance produced or imported in quantities above 10 tons per year, which is based on the traditional human health risk assessment paradigm. This involves a series of assessment steps, namely detailed physicochemical characterisation, exposure and hazard assessment, risk characterisation and uncertainty analysis [32]. 

The detailed characterisation of intrinsic physicochemical and extrinsic properties of the NBMs and medicinal products made thereof is essential to understand and predict their emissions/release and exposure, and to interpret the available toxicological data [33]. The European Chemicals Agency (ECHA) provided a recommended list of nanomaterial properties that should be measured as part of a Chemical Safety Assessment for nanomaterials [34] and the characterisation methods to measure physicochemical properties of manufactured nanomaterials [35] are generally applicable also to NBMs used in medicine.

Occupational exposure assessment starts with the identification of the possible sources of emissions/release for the activities and tasks performed by the workers or healthcare professionals, and the formulation of respective exposure scenarios that should include information on the substance, activities, route(s) of exposure, operational conditions and risk management measures (RMMs). Exposure scenarios should be defined and assessed for the synthesis phase and downstream use when NBMs are incorporated into a MD or ATMP, during the use stage when the product is applied/administered to patients by medical professionals, as well as during waste recycling, incineration and/or disposal. Occupational exposure during manufacturing processes has been already investigated for a variety of nanomaterials and can be mostly negligible for medical NBMs if appropriate RMMs are implemented. However, for the use phase of NBM-based MD and ATMP realistic occupational exposure scenarios have been formulated in the BIORIMA project. Some relevant examples include dental and surgical procedures involving the milling, drilling, grinding and polishing of materials or implants, where composites may be a source of nanoparticle inhalation exposure for dentists and surgeons [36,37,38]. Table 1 presents a list of relevant activities which may expose workers to NBMs at different life cycle stages of the MD and ATMP. 

There are a variety of approaches to assess the exposure to NBMs for the formulated exposure scenarios, which involve direct measurements or modelling. Site-specific measured data are typically preferred over model estimates and are needed to validate and improve the exposure models. Such data can be generated by portable or stationary monitoring and sampling instruments (e.g., condensation particle counter (CPC), scanning mobility particle sizer (SMPS), inductively coupled plasma-mass spectrometry (ICP-MS) or electron microscopes (SEM/TEM)) following different measurement strategies [39,40,41]. To adequately quantify the occupational exposure a multimetric approach (covering different parameters such as particle number, mass and surface-area concentrations, particle mean diameter) using a combination of instruments is recommended [42]. In the absence of measurements, exposure models for NBMs can be adapted from such models for chemicals and ENMs (e.g., NanoSafer, iEAT, Dermal Advanced REACH Tool—dART, Stoffenmanager Nano) [43,44,45,46].

Exposure assessment also requires the quantification of the bioavailable fraction that passes across the mucosal barriers (i.e., lung and intestinal epithelia). This can be estimated through experimental in vivo or in vitro testing. Moreover, using physiologically based pharmacokinetic models (PBPK) the results of rodent studies or data obtained from in vitro transport studies can be used to extrapolate a human internal exposure by simulating the absorption, distribution, metabolism, excretion of NBMs. Typically PBPK models require physiological (tissue volumes, flow rates, metabolism of chemicals, etc.), biochemical and material specific data (i.e., physicochemical properties) [47,48]. 

In general, hazard assessment of NBMs is carried out by gathering/generating and evaluating relevant physicochemical and toxicological information from in vitro and in vivo tests to assess the intrinsic hazard of a substance and to establish a dose-response relationship [49,50]. Toxicological approaches to assess hazards of NBMs can either be based on methods adopted from classical toxicology or on alternative methods, including in vitro and in vivo testing and in silico modelling (e.g., Quantitative Structure Activity Relationship (QSAR) models, grouping and read-across methods).

In fact, systems biology approaches and other advanced methods are gaining traction in the field of nanosafety research [51], though regulatory acceptance is needed in order to implement such approaches in hazard assessment of NBMs for MDs and ATMPs. ECHA identified relevant toxicological endpoints of concern for nanomaterials (e.g., cytotoxicity, inflammation, oxidative stress, genotoxicity), and a list of related appropriate in vitro and in vivo tests (e.g., in vitro mammalian cell gene mutation test, bacterial reverse mutation test) [52,53]. Furthermore, the adverse outcome pathway (AOP) framework provides pragmatic insights to promote the development of alternative testing strategies [54]. Several EU-funded projects, not least in FP7 (e.g., SUN, MARINA, NANOMILE, NANoREG projects [55]), have developed and evaluated different in vivo and in vitro protocols for investigating the hazard of nanomaterials, in principle applicable also to NBMs. These efforts have addressed the importance of assessing in vitro assays with respect to material interference [56]. Furthermore, the importance of using multiple cell types to properly evaluate the toxicity of NMs has been demonstrated in the MARINA and NANOMILE projects, and high-throughput screening approaches have been developed [57]. It is important to point out that while there is a strong (scientific and societal) incentive to move towards an animal free testing and the development of alternative test methods is highly recommended, these assays nevertheless need to be validated. The current situation however is that there are only a very limited number of validated in vitro and in silico test methods that can be used in regulatory toxicology. The exception might be the availability of in vitro (and ex vivo) approaches to evaluate acute effect on the skin. 

The importance of data management also needs to be underlined and efforts are being made to harmonise procedures for data collection and data warehousing [58]. The nano-bio community is currently debating the need for minimum information requirements when reporting research results, with the goal to improve reproducibility, increase quantitative comparisons of NMs and facilitate meta analyses and in silico modelling [59].

For any biomaterial or NBM intended for clinical use, the issue of testing for endotoxin content is of paramount importance [60]. The BIORIMA project has focused considerable efforts on the evaluation of assays for endotoxin testing, including the conventional Limulus amebocyte lysate (LAL) assay. Furthermore, the concept of the bio-corona (i.e., the adsorbed surface layer of proteins and other biomolecules) is being evaluated using biofluids that are representative of different biological/anatomical compartments. The bio-corona has received a great deal of attention in recent years in the nano-bio research field and it is known to affect the interaction of NMs with cells and tissues [25,61]. Furthermore, the bio-corona may have an impact on the targeting of NMs in relation to drug delivery or imaging [62]. However, despite these insights, the bio-corona is not yet considered in any relevant standards for NBM testing. The BIORIMA project seeks to rectify this by addressing the role of the bio-corona in hazard assessment.

#### 2.2.2. Environmental Risk Assessment

The environmental risk assessment (ERA) of NBMs contained in MDs and ATMPs is generally based on the REACH Chemical Safety Assessment, but there are prominent differences compared to chemicals, e.g., a focus on the aquatic environment with limited number of test systems [63,64]. It is well-known that NMs behave differently from ‘regular’ chemicals, in regard to environmental fate, subsequent exposure and mode of toxic action. Further, the ultimate fate of NMs is generally considered to be solid environments, like soils and sediments, rather than aquatic systems [65]. Hence, a comprehensive ERA strategy for NBMs should consider these compartments. Additional to these, there should also be a focus on sludge born materials, as sewage sludge is in many countries applied to soils [66,67]. The ecotoxicological approaches of choice to assess the hazard of NBMs can be derived from classical ecotoxicology [63] or on newer alternative approaches that provide more adequate information [68], including longer term testing [69,70,71,72] or mechanistic endpoints [73,74]. The development of alternative test methods has been highly recommended, also by regulatory agencies. For example, in the context of REACH Regulation, ECHA and European Food Safety Authority (EFSA) have proposed the use of omics data for risk assessment purposes [75]. 

The identified relevant endpoints of concern for NMs (i.e., chronic, longer term and mechanistic) and a list of appropriate testing methods [68], should in principle also be applicable to NBMs, as long as the required adaptations are included as suggested by Hund-Rinke et al., 2016 [76] and Amorim et al., 2018 [77]. For the latest update including the recommended adaptations to OECD guidelines for testing the environmental hazard of NBMs please see Amorim et al., 2020 [22]. To increase the efficiency of testing, these methods should be implemented onto IATAs, where grouping and read-across approaches are combined with testing methods and non-testing in silico models to generate data for ERA according to a tiered approach [78,79,80]. A wide number of in silico tools for NMs have been developed in recent years [81,82] and can be applied to derive data of relevance for risk assessment. Moreover, omics-techniques, including key initiating events and pathway analyses, hold great potential [70]. These techniques are highly relevant for NBMs used in MD and ATMP, as they have been developed with a specific biological purpose and therefore prior information on their mode of toxic action is available. Further, in vitro approaches in environmental organisms have also gained increasing interest, as they allow for a quick identification of relative toxicity, of possible mode of action and of what happens with a NBM when in contact with environmental media and after uptake by organisms, e.g., corona formation [83]. The latter will not only inform on possible mode of action, but also be highly relevant for supporting read-across between species. Furthermore, since MDs and ATMPs will obviously be, in most cases, in contact with human tissues, excreted NBMs will have a biological corona formed that will influence their behaviour and toxicity, and therefore their environmental risks.

As for the in situ exposure, there are advanced exposure models being developed, but these require refinement and validation especially for NBMs released from MDs and ATMPs. Therefore, the bioaccumulation/trophic transfer potential is not well understood for nanomaterials and hence also not for NBMs used in medicine. Although substantial research efforts have focused on bioaccumulation [84] there are currently no good models available, partly because nanomaterials are difficult to detect in complex media, partly because nanomaterials are not assumed to follow equilibrium paradigms, which hampers the estimation of bioaccumulation factors.

Since NBMs are synthesised and the MDs and ATMPs are produced in controlled environments, where any released NBM is adequately managed and waste is properly dealt with, it can be excluded that NBMs will reach the natural environment during synthesis and formulation, hence the life cycle stages of concern are the use and end-of-life processing and disposal. In these life cycle stages, predicted environmental concentrations (PEC) need to be estimated for relevant exposure scenarios. The detection of NBMs at trace concentrations in natural samples is in most cases not yet possible as the available analytical tools are not capable of distinguishing the NMs from natural background nanoparticles at the low NBMs concentrations expected in complex environmental matrices [85,86]. Therefore, the exposure assessment of these materials relies on environmental exposure modelling by means of material flow analysis (MFA) to predict releases from products, fate in technical systems and final release to the environment, and environmental fate models (EFM) that describe the fate of NBMs in the environment and their distribution within environmental compartments [87].

Several MFAs have been conducted for ENMs such as Ti_2_O, Ag, ZnO, SiO_2_, Al_2_O_3_, quantum dots, iron oxides, carbon nanotubes, fullerenes, using static or dynamic models assessing accumulation of ENMs in environmental compartments over several years [88,89,90,91,92,93]. So far, only two studies are available that modelled the flows of NBM to the environment. Mahapatra et al., 2015 [94] investigated the flows of nano-gold from medical applications in the United Kingdom and the United States using a bottom-up approach for the calculation of the prospective maximal consumption. Using the same approach, Arvidsson et al., 2011 [95] calculated the environmental release of nano-silver from wound dressings in Europe in a worst-case scenario. 

**Figure 2:**
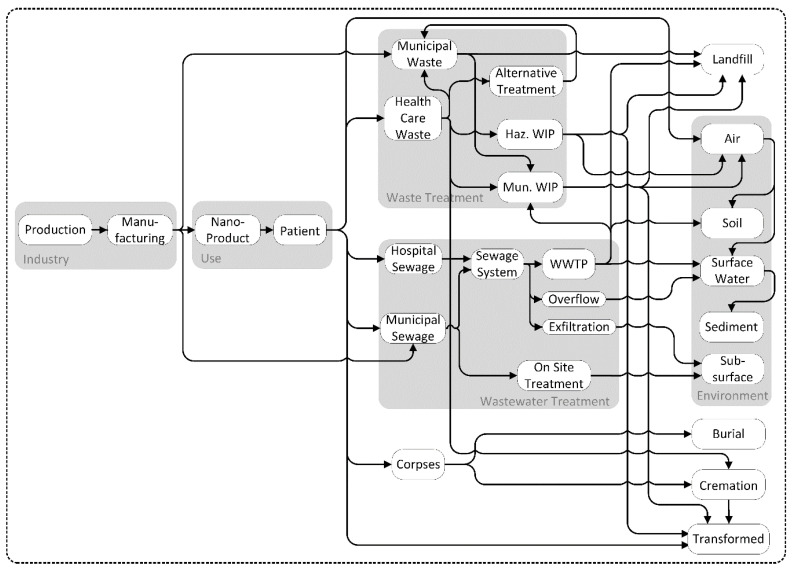
Material flow diagram for NBM from production to the intended use in patients, the wastewater and waste treatment specific to the use in hospitals and finally to the environment.

Before existing models for ENMs can be used for NBMs, several adjustments need to be made. In general, these models are also applicable to NBM as most of the parameters are based on the applications of the materials and are not particle-specific properties. A few parameters on fate in technical systems are specific to the type of particles and need to be adjusted to the type of NBM. As NBMs are mostly applied in a hospital setting, waste and wastewater from hospitals need to be included. Healthcare waste is treated differently to municipal waste due to their often-hazardous character. This means that the flows to alternative treatment or hazardous waste incinerators need to be included in the MFA model. NBMs can also be applied inside the body and stay there until the patient’s death, e.g., from their use in implants. Thus, the inclusion of crematoria or burial in cemeteries in the model is necessary. A generic model for the flows of NBM through all life-cycle stages is shown in Figure 2. The main release point is the use of the NBM in a hospital setting, with releases to waste treatment and wastewater treatment specific for the use of the investigated NBM. Depending on the type of materials (organic/inorganic) transformation can occur in several compartments such as wastewater treatment or waste incineration.

Using the environmental flow data from the MFA modelling, worst-case PECs that exclude any fate processes in the environment can be directly calculated. These values have also been referred to as ‘release concentrations’ as they do not include processes such as sedimentation or biodegradation that would decrease the environmental concentrations of the NBM. In order to consider these fate processes, EFM models developed for ENM need to be adjusted and parameterised for NBM. Such fate models are for example SimpleBox4Nano [96] or MendNano [97]. Whereas the processes that need to be considered in the fate models for NBM are the same as those for ENM, there is so far almost no data available to parameterise models for NBM. With an increased harmonisation of test protocols and test media [98] and the use of functional assays [99], also data for NBM can be obtained so that fate models can be parameterised.

The PEC values derived from MFA or EFM models can then be compared to predicted no-effect concentrations (PNECs) derived from hazard assessments. Hauser et al., 2019 [100] conducted a first environmental hazard assessment of organic and inorganic NBMs used for drug delivery based on a meta-analysis of previous ecotoxicity studies. Data are only available for a small subset of NBM, mainly for chitosan, polyacrylonitrile and hydroxyapatite. Mahapatra et al., 2015 [94] conducted the first full environmental risk assessment of an NBM, focusing on the use of nano-gold in medicine. Based on PEC values derived from MFA and a probabilistic species sensitivity distribution, it could be shown that there is no overlap between predicted exposure of nano-Au and the concentrations where adverse effects on organisms can be observed.

#### 2.2.3. Accidental Risks 

Besides chronic risks, also accidental risks for NBMs life cycle need to be considered. The focus of accidental risk assessment and management is on accidents that can result in fire, explosion or a massive release of NBMs. Smaller accidents with higher likelihood, but lower consequences, are also of interest as they can for instance impact workers handling the materials (i.e., flammability and ATEX risks). Each step of the NBMs life cycle from production, processing and use to the end-of-life is potentially bearing the risk of accidents. However, the consequences will be much higher in the first steps of the life cycle (production, transport and transformation) in which the NBMs are in high concentration and possibly in free form. Therefore, the accidental risks of these materials arising from fire, explosion and massive release phenomena need to be adequately managed based on a framework involving hazard assessment (characterisation of substances, process identification and hazardous activities); accident scenarios identification; risk assessment of the scenarios based on their likelihood and consequences. Then, depending on the acceptability level of the risks, risk management measures are identified.

To assess the risks of fire and/or explosion accidents, it is of major importance to reliably characterise the physicochemical properties, reactivity and flammability of NBMs based on established standards (CEN TS 17274:2018 [101]). This involves the application of pre-screening tools (e.g., calorimetry) and regulatory tests proposed by the United Nations Globally Harmonized System of Classification and Labelling of Chemicals (UN/GHS). Fire risks can be more specifically tested through a physical model of fire (e.g., Tewarson Fire calorimeter), while explosion risks for NBMs free forms can be assessed based on ignition sensitivity (e.g., minimum ignition energy, minimum ignition temperature, minimum explosion concentration, limiting oxygen concentration and explosion severity). These tests directly support the implementation of the ATEX Directive, and their results enable the identification of adequate safety measures.

### 2.3. Strategy for Risk Prevention and Control 

Once all risks along NBM life cycle have been assessed, adequate measures to avoid or limit/control these risks must be identified to ensure safer production, handling and disposal of NBMs. The traditional risk management of chemical substances applies to the NBMs used in medical sector. It relies on the implementation of safe-by-design and risk reduction and control measures based on the hierarchy of controls (e.g., NIOSH 2013 [102]), following the so-called STOP principle: substitution, technical measures, organisational measures and personal protection measures which can be applied throughout the life cycle of a specific material. 

#### 2.3.1. Safe by Material Design 

The elimination or substitution of hazardous constituents by means of safe-by-material design (SbMD) strategies is the first line of defence [103,104,105,106]. The SbMD approach is based on the control of nano-bio reactivity since the early stages of R&D. In a recent review Hjorth et al., 2017 [107] clearly demonstrated how the SbMD approach is inspired by safety testing and assessment practices in drug discovery and development (DDD). The authors also outlined the limitations that still delay the creation of ‘design guidelines’ for nanomaterials. The SbMD approach is based on the concept that safety is not an intrinsic property of material but can be built in through the manufacturing chain from raw materials to finished products, by adding SbMD criteria to quality assurance (QA) and good manufacturing practice (GMP) specifications.

The context and logic of implementing the SbMD idea for nanomaterials have been described in the ‘Safe-by-Design (SbD) Implementation Concept’ of the EU FP7 ProSafe project [108]. This idea builds on the SbD concept of the EU FP7 NANoREG project and the Safe Innovation Approach (SIA) of the EU H2020 NANoReg2 project. The main outputs expected by the implementation of a SbMD approach are decision criteria for selecting safer options in the early R&D steps. This requires adoption of screening-level approaches from predictive toxicology (in vitro and in silico tools) in order to speed-up the process and decrease the costs of generating material safety profiles, while taking quality and performance requirements into consideration. Indeed, as pointed out in [109] on benefit versus risk of nanomaterials: ‘Successful innovation, if it is to encompass both regulatory and consumer approval, must incorporate safety by design’.

Overall, the SbMD approach addresses safety issues at the early design stage of nano-enabled products. These issues should be formally assessed at the appropriate ‘Design Reviews’, mandatory for EMA and FDA compliance and ‘Best Practice’ for new product developments. Nevertheless, safety is not an intrinsic property of materials and the goal of safer materials can only be achieved if predictive risk assessment tools are also available that are robust and easily implementable to guide material selection and product design. These concepts are not new in both (a) drug discovery and developments and (b) ATMP where early in vitro or in silico screening is used as part of an overall risk reduction or risk mitigation strategy. It is expected that they can be successfully introduced into NBMs manufacturing processes prior to addressing detailed toxicological testing and regulation. Within the hierarchy of controls, SbMD can be allocated at the substitution level where a more hazardous material is replaced by a less hazardous material.

#### 2.3.2. Safety by Process Design 

**Figure 3:**
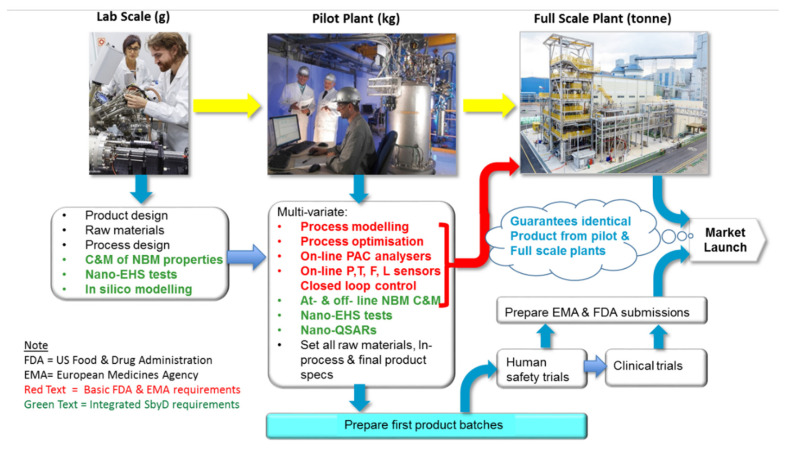
Integration of risk management framework into FDA/EMA 2003 Process Analytical Control Regulations for ATMPs (P = pressure, T = temperature, F = flow, L = level, PAC = process analytical control).

The fundamentals for safety by process design (SbPD) lay in the evolution of engineering principles initially developed in the mid-1990s for the chemical industry for manufacturing nanotitanium dioxide [110] and integrated into pharmaceutical industry manufacturing [111]. A holistic approach is adopted which starts with standard medical product GMP and QA procedures. Safety with respect to nanomaterials is built in right through the manufacturing chain from raw materials to finished products. SbPD seeks to maintain the much safer properties of the previously optimised raw materials by SbMD methods described in Section 2.3.2 throughout the production process by optimisation of all processing steps, with their respective production quantities, as described in Figure 3.

The research and development processes for in vivo medical products deploying nano- and bio- technologies differ for products that are made by materials processing (e.g., drugs and in vivo imaging agents) and by discrete product fabrication (e.g., implanted joints or devices).

SbD of medical products made by materials processing is built on the principles of the US FDA regulatory guidelines of 2003 and adopted by EMA in 2003 as described by Brenderlberger 2003 [112]. These have been further adapted for ATMPs involving NBMs within this RMF paper and described in Figure 3 below. 

Since 2003, pharmaceutical companies have adopted the FDA scheme for process analytical control (PAC) of active pharmaceutical ingredients (APIs). To control complex batch or continuous processes to manufacture API, a large number of inputs from low-functionality process sensors (e.g., pressure, temperature, flow, level and mass—P, T, F, L and M) is collected. These are linked to electrical controllers in nested hierarchical systems which may contain several distributed control systems coordinator control systems and in the case of very large manufacturing plants, super coordinator control systems. 

In PAC strategies it is essential to have specific high-information content analysers on-line at critical stages in the process. These are coupled to closed-loop control systems. Such analysers might be Fourier transform infrared spectroscopy (FTIR), Raman and infrared (IR) spectrometers, or mass spectrometers. They could also involve process tomography or automated sample and flow injection analysis through ‘lab-on-a chip’ biosensor devices or particle sizers. During the pilot plant development stage, the process parameters (P, T, F, L and M) together with reagent quantities and additives, are varied to enable the process to be optimised simultaneously for product yield, quality and process profitability. Importantly, in silico methods (e.g., nano-QSARs) are included in these calculations to minimise risks. Once optimised and tested by multivariate process modelling for the full-scale plant, all manufacturing specifications and control set points are then fixed. This guarantees that both ‘pilot’ and ‘full scale’ process plants will produce products of identical quality. These approaches collectively form the core part of safe-by-process design. It allows batches of products to be prepared for: (i) safety (‘*first in man*’ trials); (ii) clinical trials; and (iii) the EMA/FDA regulatory claims support file. This work can continue while the full-scale plant is being built and commissioned, thus shortening the time to market but guaranteeing the best possible products within a process intensification perspective [113,114].

To achieve ‘safe-by-design’ of products, additional characterisation and measurements tests and in vitro toxicology tests are added into the raw materials and pilot plant development stages (shown in green in Figure 3). These can be performed off-line or at-line using high-throughput parallel processing analysers including electron microscopy. On-line analysers are chosen for the pilot scale to enable full process analytical and product quality control. The results are analysed by nano-QSARs data analytics [115,116,117] and critical characterisation and measurement parameters together with those from the high information content on-line analysers, e.g., Raman, FTIR, mass spectrometers [110] to build multivariate statistical process control models. Together these devices are then used to maintain optimised SbD performance throughout the whole manufacturing chain from raw materials to full-scale manufacturing. 

**Figure 4:**
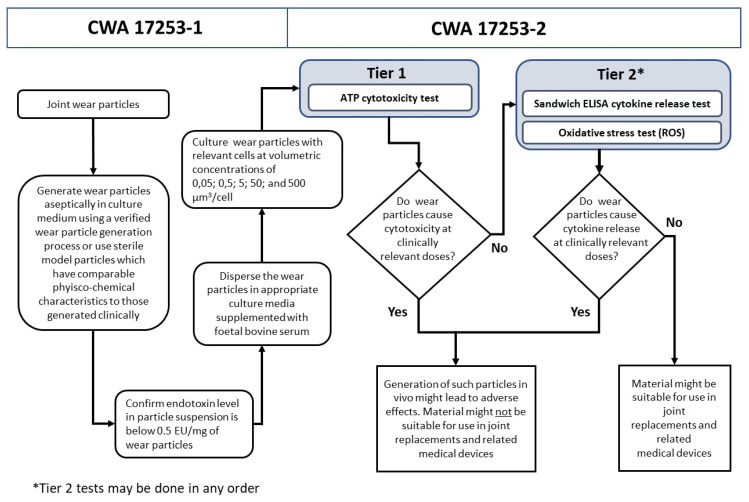
Tiered toolkit flow diagram for evaluating the biological impact of polyethylene wear particles from joint replacements and related medical devices (CWA = CEN Workshop Agreement).

Safe-by-Design for discrete object fabricated products begins with the same paradigm illustrated above for products that are made by materials processing. A significant range of materials must be tested at the raw materials stage to minimise product toxicology downstream. The approach differs at the pilot plant stage where initially material ‘test coupons’ are produced on prototype fabrication unit operations. The latter are small but mimic the automated device used in the full manufacturing plant. Critical in this early stage is to test the biocompatibility of the materials to be used for ‘implantability’ of such devices. Once suitable materials have been chosen, the implantable device geometry and performance characteristic are mathematically modelled, and the first manufacturing devices are engineered. Full-scale manufacturing for fabricated products is achieved by ‘scale out’ rather than ‘scale up’. The imperative is to ensure the unit operations are fully optimised. These are then replicated to create a manufacturing line with banks of identical unit operations accurately producing the product in parallel to a full set of specifications, including NBM hazard reduction. Using nano-enabled SbD replacement hip joints, this generic SbD process development has been described for the first time in the BIORIMA project by Wilkins and colleagues and is illustrated in Figure 4 below. 

As part of the manufacturing fabrication development, product performance is assessed by simulation and accelerated mechanical testing for in vivo use. In addition, these authors noted that the standards issued by the International Organization for Standardisation (ISO) and the European Committee for Standardisation (CEN) addressed only the risks of bulk materials of devices and not the risk of NBM used in the devices or created during lifetime wear. They have set a benchmark for such standards through CEN Workshop Agreements (CWA), CWA 7253-1:2018 and CWA 17253-2:2018, respectively for NBM wear characterisation and toxicology testing, to be applied to all nano-enabled replacement joint devices.

The above same principles can also be applied to personalised medicine applications such as patient-specific 3D printed implants, containing nanohydroxyapatite to replace bone loss, following maxillary facial surgery to remove bone affected by cancer [118].

#### 2.3.3. Risk Reduction and Control

In many cases it is not possible to substitute a specific substance and/or process and thus the further measures have to be applied during production and use. For risk reduction and control during the production phase, first of all targeted and well-defined technical measures (engineering controls applying closed processes, fume hoods, enclosed glove boxes, etc.,) have to be applied [104]. These have to be process and material specific and should be supported by on-site measures that may decline or support an initial suspicion of material release. If a measurement does not reveal any release during normal processing (e.g., higher concentration than the background and meeting the legal restrictions) no further acute action is needed. Nevertheless, the technical measures should be conceived as far as possible to also cover accidental scenarios (cf. Section 2.2.3). Organisational measures include administrative controls like operational procedures such as HEPA (High-efficiency particulate air)-filtered vacuum cleaning, regular wet wiping of surfaces and equipment but also periodical checks on the effectiveness of these procedures and training of the involved personnel. Further risk reduction and control is achieved by (additionally) using personal protective measures, that is equipment like eye protection, gloves and respirators with different adjusted protection levels. Furthermore, discontinuous control measures as well as a continuous exposure monitoring on site (see the next paragraph) with appropriate measurement equipment for identification and quantification might be needed. 

The above described hierarchy of controls is also applicable during the use phase, at least for professionals working with the NBMs. It then has to be modified to cope for the material and use specific properties in case of medical devices (e.g., scaffolds, implants) or the direct application of materials (e.g., food supplements, medicine, etc.,): (i) Engineering controls can minimise abrasion, dissolution and local exhaust during mechanical treatments; (ii) administrative control can involve operational procedures for preparation and application of ATMP, specific cleaning procedures, waste handling, training of involved personnel; (iii) personal protective equipment can involve the use of gloves and face masks by healthcare staff, while for patients use of tissue barriers, direct removal of debris, etc. In addition, health surveillance (continuous medical supervision) might be needed. Employees working with NBMs should be informed, trained and supervised regularly. 

#### 2.3.4. Benefit-Risk Analysis for Managing Risks for Patients

Innovative MD and ATMP containing NBMs could address current unmet therapeutic or diagnostic needs, however they may be developed, manufactured and used in completely new ways compared with conventional medicinal technologies and this can challenge their market authorisation. Therefore, to properly support the translation of NBMs used in MD and ATMP into clinical use, careful assessment of their benefit-risk balance is required [119] and this task is covered by the benefit-risk analysis component of the RMF (right-side pillar, cf. Figure 1). 

When assessing the use of NBMs in ATMP and MD, it is important to underline that they can pose specific regulatory challenges related to their inherently complex nature and their relative novelty in the medical field. These materials involve complex nanostructures that can possibly trigger a wide range of biological responses. The broadly adopted ‘conventional’ approaches for physicochemical and toxicological testing of medicinal products have been accepted also for nanoparticle medicinal products [120]. However, it has been acknowledged that these methods are not yet fully adapted to address the inherent complexity of these nano-bio systems, which raises concerns about how reliable is the current dataset for regulation [120]. 

In simplified terms benefit risk analysis is performed to answer the question—do the benefits of a NBM outweigh the risks to the target individual or population and are the uncertainties reasonably low [121]? The answer to this question is important for both the industry developing these NBMs and the regulatory authorities. Benefit risk analysis is generally performed when applying for market approval for a medicine following clinical trials, however it has been recognised that the approach should be performed throughout the whole R&D phases of the medicinal product, including the non-clinical discovery phase, the clinical phases (phase I, II, III) and the post-marketing pharmacovigilance [19,20]. 

In order to carry out a benefit risk analysis according to regulatory requirements, a review of the scientific data should be performed, taking into account both benefits and risks of a given NBM for the target population [122,123]. Figure 1 (right side) describes this task and depicts the main steps of a benefit risk analysis. 

First, the results of the decision context, encompassing the analysis of the therapeutic context, the available comparators, the horizon and the stakeholder perspectives [124] provide suitable information to properly identify the benefits and risks associated to the specific treatment. Benefits and risks are then weighted and compared in order to evaluate if the benefits outweigh risks. When considering benefit risk analysis, it is important to have consistent definitions of the related terms. Here ‘benefit’ relates to a favourable outcome (e.g., increased efficacy) of a given medical application, while ‘risk’ is used to denote adverse effects defined by severity and probability of occurrence [122,125]. In contrast to occupational or environmental risks, which are calculated as absolute quotients that are strictly acceptable or non-acceptable, the risks from MD or ATMP are always relative to the expected therapeutic benefits and to the potential consequences the specific health problem can bring to the patients (e.g., death, impairment). Therefore, for medical applications such as MD and ATMP, the estimation of the dose-response relationship of possible adverse effects needs to be coupled with a benefit risk analysis, which considers additional criteria such as the nature and severity of the disease to be treated, the possible benefits of the treatment to the patient and the levels of risk acceptance on both the community (societal) and patient (personal) levels. For both benefit and risk, it is recommended that uncertainties such as variation, methodological flaws or deficiencies unsettled issues, limitations of the data set be considered during benefit risk analysis [126]. IATA, as already discussed in Section 2.2.1, can support the analysis of existing information available along the product development phases, with the aim of guiding the selection of the most suitable and effective tests to provide the information needed to perform an effective benefit risk analysis.

Benefit risk analysis can be adapted considering the R&D phase of the MD or ATMP. Indeed, risks can be detected in non-clinical phase and continue throughout the development of the MD or ATMP in order to prevent and minimise risks when possible [127]. 

In the non-clinical discovery phase, also known as Go/No-Go decision, a MD or ATMP containing NBMs needs to pass through several steps, which include determination of drug availability, absorption, distribution, metabolism and elimination and preliminary studies to investigate safety aspects such as genotoxicity, mutagenicity, safety pharmacology and general toxicology [128]. Moreover, in this phase, the application of in silico and in vitro tests, complemented by ex vivo and in vivo assays (if necessary) can help to recognise safety/toxicity issues early in the process to correct those prior to the final selection of clinical candidates [19]. In the pre-clinical phase, information of the disease obtained from animal models are compared with data from toxicological studies to determine whether (or not) a candidate type of NBM can be administered for the first time in humans [20]. In this phase, hypothetical benefits are assessed based on current understanding of the mode of action for the NBM identified in animal or in vitro tests, along with a nonclinical and in vitro safety evaluation [129]. During the clinical development, the registration process and the marketing period, benefit risk analysis involves identifying potential efficacy and safety endpoints and other surrogates as well as a more precise safety profile and the identification of adverse effects.

According to the ATMP guideline [127], after the development of a benefit risk analysis, the applicant should provide a risk management plan (RMP) for obtaining the market authorisation. In the RMP, safety specification, pharmacovigilance plan and risk minimisation activities need to be assessed. Safety specifications consists in the identification of risks to be minimised and/or characterised during the post-marketing phase considering risks derived from the product manufacturing, handling, application and clinical follow-up (i.e., risks to patients due to interaction with other medicinal products or maladministration and risks to healthcare professionals). Pharmacovigilance activities consist in the identification, quantification and characterisation of safety hazard and the measurement of effectiveness of risk-management measures, while the risk minimisation plan includes risk minimisation measures such as supplement information about conditioning of the patient, product characteristics, adverse drug reactions, healthcare professional protection measures. Safety and efficacy (S&E) follow-up data need to be provided to support the marketing authorisation application considering the ATMP characteristics and its intended indication, while long-term S&E follow-up activities are only related to ATMP. 

After the clinical studies of a MD, a risk management file must be developed for the post-market surveillance, including results from the risk-benefit analysis and risk minimisation measures. The risk management file should contain definitions of possible hazardous situation associated to the use of the investigated MD and all the possible applicable risk minimisation measures for patients as well as healthcare personnel. This includes the risk analysis, risk evaluation, the implementation and verification of the risk control measures and the final assessment of the acceptability of residual risks [130].

In 2009, EMA established a 3-year research program whose purpose was ‘to develop and test tools and processes for balancing multiple benefits and risks as an aid to informed regulatory decisions about medicinal products’ [129]. According to the results of this study, the prominent form of benefit risk analysis framework is multicriteria decision analysis (MCDA), although these have been mostly academic to date. The EMA has participated in the development of MCDA frameworks such as PrOACT-URL (problem formulation, objectives, alternatives consequences, trade-offs, uncertainties, risk tolerance) [121]. Additionally, the pharmaceutical industry has also developed benefit-risk assessment such as the PhRMA BRAT framework (Pharmaceutical Research and Manufacturers of America, the Benefit-Risk Action Team), a six step process that focuses on documenting rationale for decisions [124]. However, ideally a systematic, transparent and structured regulatory decision-making process is required that is of use to all stakeholders. The development of the UMBRA (Universal Methodology for Benefit-Risk Assessment) framework makes steps towards this structured regulatory decision-making process as it incorporates several frameworks (PrOACT-URL, PhRMA BRAT and FDA 5-step framework) [131]. The UMBRA framework uses benefit-risk summary template and corresponding user manual to clearly communicate benefit risk analysis to all stakeholders and upon review was found to be of value by several regulatory agencies [132,133].

As far as MD are concerned, the use of relevant harmonised standards is required to demonstrate conformity with the general safety and performance requirements and other legal requirements, such as those relating to quality and risk management [134,135,136]. A benefit-risk ratio needs to be estimated, which requires that all known and foreseeable risks shall be minimised and weighed against the evaluated benefits to the patient and/or user of the MD during normal conditions of use. 

However, as pointed out by Halamoda-Kenzaoui et al., 2019 [10], for some endpoints such as drug release/loading and the interaction of nanomedicines with the immune system no standards are available so far. This creates a potential Catch-22 situation inasmuch as the anticipation of standardisation needs require a good understanding on the regulatory information for nanomedicines while, on the other hand, robust datasets allowing firm conclusions in regard to regulatory demands are not yet available [10]. Efforts are currently being made across several EU-funded projects including BIORIMA to develop robust test methods for hazard assessment of NBMs to set the stage for standardisation of NBMs in MD and ATMP while the REFINE (Regulatory Science Framework for Nano(bio)material-based Medical Products and Devices) project recently issued a report to highlight regulatory needs in nanomedicine [10].

To properly identify, assess and manage risks, ECHA along with the FDA, Health Canada, Australia Therapeutic Goods Administration, Japan Ministry of Health, Labour and Welfare (MHLW) endorsed ISO 14971:2007 [137]. ISO 14971 applies only to manufacturers placing MD on the market in Europe, since it introduced three new annexes (ZA, ZB and ZC Annexes) specifically developed to align with the EU MD directives. It is expected that revised versions of these annexes will be soon available to include the requirements of the new EU Medical Device Regulation [16]. 

## 3. Perspectives on the Implementation of the Risk Management Framework

The BIORIMA RMF has been developed to be flexible and efficient [138]. It is flexible enough to address different assessment goals depending on user needs. It is efficient in collecting information for risk assessment based on specific user goals (i.e., targeted testing) by means of optimal IATA, instead of fulfilling predefined data requirements. This is intended to ensure an optimal balance between compiling the data needed for a targeted and accurate risk assessment and for selecting adequate risk control measures, and the efforts and cost required to collect these data. 

In addition to the safety for patients, the occupational and ecological risks from NBMs used in medical applications need to be thoroughly assessed and managed. For example, the extensive use of nano-Ag in biomedical applications (e.g., wound dressing, catheters) is motivated by the increased antimicrobial activity if compared to the bulk form, but toxicity and inflammatory response in humans need to be controlled, and the possible contribution to silver resistance in bacteria in the long term raises concerns [139]. 

If the assessment of risks for different targets can be considered adequately addressed through distinct regulations, there is a need for a scientific framework and guidance on the best experimental and modelling approaches to do it. For instance, in absence of sufficient environmental monitoring data, the application of material flow modelling could help investigating the expected concentrations of nano-Ag in different environmental compartment based on average production data [87].

The BIORIMA RMF aims to help industry in the fulfilment of regulatory requirements and, at the same time, in the development of safer NBM applications while retaining their efficacy, performance and quality, so that they can successfully enter the market. It will provide guidance to identify the specific regulatory requirements in each step of the supply chains of these products and will suggest how to address them by means of appropriate safety testing and assessment strategies based on the state-of-the-art scientific knowledge. 

It should be recognised that the benefit risk analysis process is required for the market authorisation of a new MD or ATMP, and the process of assessing occupational and environmental risks (e.g., REACH or Environmental Health and Safety regulations) are inherently different and require distinct strategies. This is not only because of the different regulatory regimes, but also because the very concept of ‘risk’ and its perception and acceptability are different in these two areas. The risks for workers and the environment are determined by unintentional exposure to the materials, they are absolute in nature, assessed based on conservative assumptions and any risks above the exposure/hazard risk ratio are considered unacceptable. In contrast, the risks for patient posed by medical applications are always compared to their clinical benefits and can be accepted if the benefits significantly outweigh the safety concerns. These fundamental differences have determined different requirements in the respective regulations that require different testing strategies. However, some of the (standard) testing methods, modelling tools and data can be used across these domains. The implementation of the RMF can facilitate this by identifying areas of cross-fertilisation to promote sharing of ideas, data and tools. In this sense, the RMF can promote and facilitate the communication and collaboration between scientists from different fields.

One important area of cross-fertilisation between the assessment of risks for workers, patients or the environment is physicochemical characterisation. In this area the RMF will offer guidelines for testing of both intrinsic and extrinsic properties as the relevant standards are still in early stages of development [140] while this is crucial baseline information on how to proceed with the evaluation of potential risks. 

Moreover, guidelines are needed also to integrate the outcomes of physicochemical characterisation with (eco)toxicological assessment. Therefore, the RMF provides such guidance in the form of a set of structured IATA, presented as decision trees to guide the selection of in vitro and in vivo tests based on material identity. The outlook is to deliver IATA through a software-based BIORIMA Decision Support System for risk assessment and management of NBMs used in MDs and ATMPs, that will be freely accessible on-line to end-users. 

The adoption of a life cycle approach in the assessment of occupational and environmental risks is a key element as in the formulation and use stages significant exposure of workers and healthcare staff could occur if there are no adequate risk management measures in place.

For example, the use of hydroxyapatite-based dental composite in the dental sector requires the development of tailored occupational exposure scenario. This paste can be grinded, polished or shaped during the application by the dentist, who could be exposed to airborne nanoparticles if not adequately protected [37]. This and similar exposure scenarios involving processing of artificial joints during replacement surgeries are of potentially high concern and their possible risks should be investigated, especially in the case the relevant exposure routes are different from those already assessed for intentional administration/use of the products by patients. 

Healthcare professional can therefore benefit from the implementation of the RMF in terms of increased awareness about potential occupational risks and improved knowledge of adequate prevention and control measures. To develop an effective risk prevention and control strategy it is important to clearly define the target groups of concern for each specific intended use of the NBM (e.g., doctors, dentists, surgeons, nurses) and train them in working with these substances and using the relevant safety measures, especially in view of personal protective equipment, which is only effective if properly used. The known level toxicity of the potentially released NBMs is a key starting point to determine the needed level of controls. Moreover, such a strategy should be based on reliable onsite measurements of emissions/release and exposure. Even though some processes might be evaluated by lab-scale simulations, the complexity and maybe also uniqueness of a specific workplace requires exposure measurements based on a mixture of personal and area sampling using appropriate equipment and including background measurements. These exposure measurements can then provide guidance for the evaluation of risk management measures and their effectiveness, which should consider specific characteristics of the healthcare sector, such as the transfer of NBMs from high security areas to uncontrolled areas or waste management practices where day-by-day exposure to NBMs is possible to occur. The effectiveness of protection measures already in place in hospitals (e.g., for handling of cancer treatment medicine) should be carefully evaluated and similar measures should also be adopted for NBM-based MDs and ATMPs. In other words, a read-across from the handling of other hazardous substances like anti-cancer drugs, biological agents and/or radioactive materials can be beneficial to set the frame for handling of potentially less toxicologically potent NBMs. 

In the use phase, to ensure patient safety, benefit risk analysis should be performed when progressing to in vivo pre-clinical and clinical trials and should be benchmarked against current treatment for the same disease. Such an analysis could also be performed in the pre-commercial steps, during the early stages of the innovation process when new NBM-enabled applications are developed and there is the need to understand the balance of their anticipated clinical benefits and possible safety implications.

In this context, the RMF could also represent a useful tool to improve the communication to patients about evidence on risks and benefits and how they are weighted and judged. There is indeed a growing awareness of the role that active patients and public participation can play in improving decision-making on health technologies, that would eventually help improving adherence to treatments [141]. 

## 4. Conclusions

Here we provided an overview of the BIORIMA risk management framework (RMF) in conceptual terms. The RMF has been designed to be applicable to past and current generations of NBMs, while at the same time integrate new scientific insights to address the innovation and regulation needs of future NBM generations. The RMF has adopted a life cycle approach for risk assessment and management not only for patients intentionally exposed to NBM-based MDs and ATMPs in the use phase, but also for workers and healthcare professionals and the environment that may be accidentally exposed to NBMs released in the synthesis, product manufacturing, use and end-of-life stages. The RMF provides a flexible and efficient approach to address different assessment goals depending on user needs in order to find the optimal balance between compiling the data needed for a targeted and accurate risk assessment, for selecting adequate risk control measures and the efforts and cost required to collect these data. This is achieved by means of so-called IATAs designed to guide stakeholders (e.g., manufacturers, regulators, scientists, consultants) in identifying the most efficient test cascade to generate the data necessary for risk assessment and management. The RMF addresses the differences in requirements in the chemicals and medical regulatory domains and facilitates cross-fertilisation for exchange of ideas, data and (standard) testing methods and modelling tools between these areas. The next steps are to operationalise the conceptual RMF and deliver it both as a guidance and a software-based DSS for risk assessment and management of NBMs used in MDs and ATMPs. 

## Figures and Tables

**Table 1:** Examples of occupational exposure routes and targets for tasks/activities identified within life cycle of NBMs used in MD and ATMP.

Type of Product	Life Cycle Stages	Tasks/Activities	Exposure Route	Target
ATMP	Synthesis of NBMs	Weighing operations Mixing operations Purification Collection and sorting Packing/re-packing	Inhalation Dermal	Workers in medical research labs
	Product manufacturing	Dissolution preparation Sampling Packing/re-packing In vitro and In vivo testing Cleaning and maintenance Waste management Collection and sorting Packing/re-packing	Inhalation Dermal	Workers in medical research labs Workers in pharma industry Facility maintenance staff
	Use	Flask filling and mixing operation Syringe filling (1–60 mL) Maintenance of drug preparation devices Waste management	Inhalation Dermal	Health care workers Home healthcare workers Waste management workers
	End of Life	Handling patient excreta Spills treatments Waste management	Inhalation Dermal	Health care workers Waste management workers
MD	Synthesis of NBMs	Weighing operations Mixing operations Purification Collection and sorting Packing/re-packing	Inhalation Dermal	Workers in medical research labs
	Product manufacturing	NBMs dosage Injection molding Machining and abrasion Mixing operations Film coatings Collection and sorting Packing/re-packing	Inhalation Dermal	Workers in medical research labs Workers in pharma industry Facility maintenance staff
	Use	Drilling of tooth Polishing and sanding Application in the body Waste management	Inhalation Dermal	Health care workers Home healthcare workers Waste management workers
	End of Life	Handling patient excreta Waste management	Dermal	Waste management workers

## Supplementary Materials

The following are available online at https://www.mdpi.com/1996-1944/13/20/4532/s1. Definitions of Biomaterial, Nano-biomaterial, Medical Device, Advanced Therapy Medicinal Products. Table S1: Types of industries producing and/or using NBMs as well as healthcare bodies where NBM-based MD or ATMP can be used (not exhaustive list), Table S2: List of participants to the 1st BIORIMA Stakeholder Workshop held in Valencia (Spain) in November 2018 (the names and complete affiliation of the representatives are not provided for personal data protection and privacy reasons).

## Abbreviations

AOP	Adverse Outcome Pathway
API	Active Pharmaceutical Ingredient
ATEX	Explosive Atmospheres
ATMP	Advanced Therapy Medicinal Products
CPC	Condensation Particle Counter
CWA	CEN Workshop Agreement
DDD	Drug Discovery and Development
ECHA	European Chemicals Agency
EFM	Environmental Fate Models
EFSA	European Food Safety Authority
EMA	European Medicines Agency
ENM	engineered nanomaterial
ERA	environmental risk assessment
FDA	Food and Drug Administration
FTIR	Fourier transform infrared spectroscopy
GMP	Good Manufacturing Practice
IATA	Integrated Approaches to Testing and Assessment
ICP-MS	Inductively Coupled Plasma-Mass Spectrometry
LAL	Limulus amebocyte lysate
MCDA	Multicriteria Decision Analysis
MD	Medical Device
MFA	Material Flow Analysis
MHLW	Ministry of Health, Labour and Welfare
NBM	nano-biomaterial
OECD	Organisation for Economic Co-operation and Development
PAC	Process Analytical Control
PBPK	Physiologically Based Pharmacokinetic
PEC	Predicted Environmental Concentrations
PhRMA BRAT	Pharmaceutical Research and Manufacturers of America, the Benefit-Risk Action Team
PNEC	Predicted No-Effect Concentrations
PrOACT-URL	Problem formulation, Objectives, Alternatives Consequences, Trade-offs, Uncertainties, Risk tolerance
QA	Quality Assurance
QSAR	Quantitative Structure Activity Relationship
REACH	Registration, Evaluation, Authorisation and restriction of Chemicals
RMF	Risk Management Framework
RMM	Risk Management Measures
RMP	Risk Management Plan
R&D	Research and Development
SbD	Safe-by-Design
SbMD	safe by material design
SbPD	safety by process design
S&E	Safety and Efficacy
SEM	Scanning Electron Microscopy
SMPS	Scanning Mobility Particle Sizer
TEM	Transmission Electron Microscopy
UMBRA	Universal Methodology for Benefit-Risk Assessment
UN/GHS	United Nations Globally Harmonized System of Classification and Labelling of Chemicals
