# Supplementary material for: Risk Management Framework for Nano-Biomaterials Used in Medical Devices and Advanced Therapy Medicinal Products
Source: Materials (Basel). 2020 Oct 13;13(20):4532. doi: 10.3390/ma13204532 (PMC7601697; doi:10.3390/ma13204532)
Supplement: Supplementary file 1 [file materials-13-04532-s001.pdf]

materials

MDPI

Supplementary

Risk Management Framework for Nano-Biomaterials Used in Medical Devices and Advanced Therapy Medicinal Products

Elisa Giubilato [1], Virginia Cazzagon [1], Mónica J. B. Amorim [2], Magda Blosi [3], Jacques Bouillard [4], Hans Bouwmeester [5], Anna Luisa Costa [3], Bengt Fadeel [6], Teresa F. Fernandes [7], Carlos Fito [8], Marina Hauser [9], Antonio Marcomini [1], Bernd Nowack [9], Lisa Pizzol [10], Leagh Powell [7], Adriele Prina-Mello [11], Haralambos Sarimveis [12], Janeck James Scott-Fordsmand [13], Elena Semenzin [1], Burkhard Stahlmecke [14], Vicki Stone [7], Alexis Vignes [4], Terry Wilkins [15], Alex Zabeo [10], Lang Tran [16] and Danail Hristozov [1,*]

1 Department of Environmental Sciences, Informatics and Statistics, University Ca' Foscari of Venice, Via Torino 155, 30172 Venice, Italy; giubilato@unive.it (E.G.); virginia.cazzagon@unive.it (V.C.); marcom@unive.it (A.M.); semenzin@unive.it (E.S.)
2 Department of Biology and CESAM, University of Aveiro, 3810-193, Aveiro, Portugal; mjamorim@ua.pt
3 Institute of Science and Technology for Ceramics, National Research Council of Italy (CNR-ISTEC), Via Granarolo 64, 48018 Faenza, Italy; magda.blosi@istec.cnr.it (M.B.), anna.costa@istec.cnr.it (A.L.C.)
4 Institut National de l'Environnement industriel et des Risques, Parc Technologique ALATA, 60550 Verneuil-en-Halatte, France; Jacques.bouillard@ineris.fr (J.B.), alexis.vignes@ineris.fr (A.V.)
5 Division of Toxicology, Wageningen University, 6708 WE, Wageningen, The Netherlands; hans.bouwmeester@wur.nl
6 Division of Molecular Toxicology, Institute of Environmental Medicine, Karolinska Institutet, 171 77 Stockholm, Sweden; bengt.fadeel@ki.se
7 Heriot-Watt University, Edinburgh EH14 4AS, UK; T.Fernandes@hw.ac.uk (T.F.F.), l.powell@hw.ac.uk (L.P.), v.stone@hw.ac.uk (V.S.)
8 Instituto Tecnologico del Embalaje, Transporte y Logistica, 46980 Paterna - Valencia, Spain; carlos.fito@itene.com
9 Empa, Swiss Federal Laboratories for Materials Science and Technology, Lerchenfeldstrasse 5, 9014 St. Gallen, Switzerland; Marina.Hauser@empa.ch (M.H.), nowack@empa.ch (B.N.)
10 GreenDecision Srl, Via delle Industrie, 21/8, 30175 Venice, Italy; lisa.pizzol@greendecision.eu (L.P.); alex.zabeo@greendecision.eu (A.Z.)
11 Trinity Translational Medicine Institute, Trinity College, The University of Dublin, Dublin 8, Ireland; prinamea@tcd.ie
12 School of Chemical Engineering, National Technical University of Athens, Athens 15780, Greece; hsarimv@central.ntua.gr
13 Department of Bioscience, Aarhus University, 8600 Silkeborg, Denmark; jsf@bios.au.dk
14 Institut für Energie und Umwelttechnik e.V., 47229 Duisburg, Germany; stahlmecke@iuta.de
15 Nanomanufacturing Institute, School of Chemical and Process Engineering, University of Leeds, Leeds LS2 9JT, UK; t.a.wilkins@leeds.ac.uk
16 Institute of Occupational Medicine, Research Avenue North, Riccarton, Edinburgh EH14 4AP, UK; lang.tran@iom-world.org
* Correspondence: danail.hristozov@unive.it

1. Definitions

A **Biomaterial**, according to the definition of the American National Institute of Health, can be described as any substance or combination of substances, other than drugs, synthetic or natural in origin, which can be used for any period of time, which augments or replaces partially or totally any tissue, organ or function of the body, in order to maintain or improve the quality of life of the individual [1].

A **Nano-biomaterial** is defined as a special category of biomaterials that contain a constituent or have a surface size in the nano range (i.e., between 1 and 100 nm) [2].

A **Medical Device (MD)** is defined, according to Regulation 2017/745/EC as any instrument, apparatus, appliance, software, implant, reagent, material or other article intended to be used on humans for the diagnosis, prevention, monitoring, prediction, prognosis, treatment, alleviation of disease or compensation for, an injury or disability, investigation, replacement or modification of the anatomy; conception control by mechanical or physical means; examination of specimens derived from the human body and products specifically intended for the cleaning, disinfection or sterilisation of devices. A medical device should not achieve its principal intended action by pharmacological, immunological or metabolic means, in or on the human body, but may be assisted in its function by such means.

A MD can have one or more of the following intended uses (ISO 10993-1:2009 [3]):

- Surface contacting devices: MD that comes into contact with skin. Examples are wound dressings containing nano-sized silver particles and metal oxide particles used for improved antibacterial and anti-fungal activity.
- External communicating devices: MD that comes into contact with the blood path, either indirectly or with circulating blood, and devices in contact with tissue/bone/dentin. Examples include catheters with a nanosilver coating, polymer-based dental composite filler materials and dental cements containing nanoparticles, surgical and dental instruments with nano-coatings structures used to enhance the wear resistance or to create non-sticky surfaces.
- Implant devices: MDs which are intended to be totally introduced into the human body, are in contact with tissues, bone or blood, and are intended to remain in place after the procedure. Examples include nanocoated bare metal stents, implants for joint replacement (arthroplasties) and for fracture repair, surface nano-coatings on implants used to improve the biocompatibility or for antibacterial purposes, carrier material ('scaffold') for tissue engineering products with a nanoporous structure and surface properties that facilitate the growth of living cells and enabling the tissue of replace, repair or regenerate tissues (e.g., bone fillers with hydroxyapatite and tricalcium phosphate nanoparticles, carbon nanotubes in bone cements).

Advanced Therapy Medical Products (ATMPs) constitute a class of innovative pharmaceuticals based on emerging cellular and molecular biotechnologies, encompassing the following typologies:

- Gene therapy medicinal products (as defined in Part IV of Annex I to Directive 2001/83/EC, as amended): Medicines that contain genes that lead to a therapeutic, prophylactic or diagnostic effect. They work by inserting 'recombinant' genes into the body, usually to treat a variety of diseases, including genetic disorders, cancer or long-term diseases. A recombinant gene is a stretch of DNA that is created in the laboratory, bringing together DNA from different sources.;
- Somatic cell therapy medicinal products (as defined in Part IV of Annex I to Directive 2001/83/EC, as amended): Medicines containing cells or tissues that have been manipulated to change their biological characteristics or cells or tissues not intended to be used for the same essential functions in the body. They can be used to cure, diagnose or prevent diseases;
- Tissue engineered products (as defined in Article 2(1)(b) of Regulation (EC) No. 1394/2007): Medicines that contain cells or tissues that have been modified so they can be used to repair, regenerate or replace human tissues.

Moreover, tissue or cell can be associated to a medical device as an integral part of the product and in this case, we refer to combined ATMPs (e.g., cells embedded in a biodegradable matrix or scaffold), which can fulfil any of the above-mentioned intended uses.

2. List of Industries and Healthcare Facilities

Table S1. Types of industries producing and/or using NBMs as well as healthcare bodies where NBM-based MD or ATMP can be used (not exhaustive list).

**Development and production of NBMs or NBM-based medical devices and ATMP**	Pharmaceutical industries
	SMEs producing NBMs/Medical Devices/ATMPs
	Contract Research Organization (CROs)
	Academic research centres and laboratories
	Research hospitals
**Use of NBM-based medical devices or ATMP**	Hospitals
	Clinical centres
	Dental clinics
	Urgent care centres
	Hospice centres
	Rehabilitation centres
	Imaging and radiology centres

3. List of Stakeholders—Workshop Valencia 2018

Table S2. List of participants to the 1st BIORIMA Stakeholder Workshop held in Valencia (Spain) in November 2018 (the names and complete affiliation of the representatives are not provided for personal data protection and privacy reasons).

Delegate	Affiliation	Country
1	Academia	Portugal
2	Research	Spain
3	Academia	Spain
4	Academia	Spain
5	Industry	Spain
6	Industry	Spain
7	Industry	Italy
8	Research	Spain
9	Industry	Italy
10	Regulator	Belgium
11	Industry	Italy
12	Academia	Italy
13	Industry	Spain
14	Regulator	Germany
15	Industry	Spain
16	Industry	Italy
17	Academia	Italy
18	Industry	Spain
19	Industry	Spain
20	Academia	Spain
21	Academia	Spain
22	Industry	Spain
23	Research	Italy
24	Academia	Japan
25	Academia	Greece
26	Research	Spain
27	Academia	Spain
28	Research	Spain
29	Research	Greece

30	Research	Italy
31	Research	Spain
32	Academia	UK
33	Academia	Irelan
34	Industry	Italy
35	Research	Austria
36	Industry	Germany
37	Industry	Spain
38	Industry	Spain
39	Industry	USA
40	Academia	Danemark
41	Research	Italy
42	Research	Germany
43	Industry	UK
44	Research	UK
45	Regulator	The Netherlands
46	Academia	Spain
47	Research	France
48	Industry	Germany
59	Academia	UK

References

1. Williams, D.F. *The Williams Dictionary of Biomaterials*; Liverpool University Press: Liverpool, UK, 2011; doi:10.5949/UPO9781846314438.
2. Yang, L., Zhang, L., Webster, T.J. 2011. Nanobiomaterials: State of the art and future trends. *Adv. Eng. Mater.* 13, 197–217. https://doi.org/10.1002/adem.201080140
3. International Organization for Standardization (ISO). *ISO 10993-1:2009. Biological Evaluation of Medical Devices. Part 1: Evaluation and Testing*; ISO: Geneva, Switzerland. 2009.

© 2020 by the authors. Submitted for possible open access publication under the terms and conditions of the Creative Commons Attribution (CC BY) license (http://creativecommons.org/licenses/by/4.0/).
